# Evolution of female carotenoid coloration by sexual constraint in *Carduelis *finches

**DOI:** 10.1186/1471-2148-10-82

**Published:** 2010-03-25

**Authors:** Gonçalo C Cardoso, Paulo Gama Mota

**Affiliations:** 1 Department of Zoology, University of Melbourne, Melbourne, VIC 3010, Australia; 2 CIBIO, Centro de Investigação em Biodiversidade e Recursos Genéticos, Campus Agrário de Vairão, 4485-661 Vairão, Portugal; 3 Departamento de Ciências da Vida, Universidade de Coimbra, 3000-056 Coimbra, Portugal

## Abstract

**Background:**

Females often express the same ornaments as males to a similar or lesser degree. Female ornaments can be adaptive, but little is known regarding their origins and mode of evolution. Current utility does not imply evolutionary causation, and therefore it is possible that female ornamentation evolved due to selection on females, as a correlated response to selection on males (sexual constraint), or a combination of both. We tested these ideas simulating simple models for the evolution of male and female correlated traits, and compared their predictions against the coloration of finches in the genus *Carduelis*.

**Results:**

For carotenoid-based ornamental coloration, a model of sexual constraint on females fits the *Carduelis *data well. The two alternative models (sexual constraint on males, and mutual constraint) were rejected as causing the similarities in carotenoid coloration between males and females. For melanin coloration, the correlation between the sexes was weaker, indicating that males and females evolved independently to a greater extent.

**Conclusions:**

This indicates that sexual constraint on females was an important mechanism for the evolution of ornamental carotenoid coloration in females, but less so for melanin coloration. This does not mean that female carotenoid coloration is non-adaptive or maladaptive, because sexual dichromatism could evolve if it were maladaptive. It suggests, however, that most evolution of female carotenoid coloration was male-driven and, when adaptive, may not be an adaptation *stricto sensu*.

## Background

Sexual ornaments are often expressed in males and females of the same species, to the same or different degrees. It is well established that male ornaments evolve mainly due to sexual selection [1]. Female ornaments were previously thought to be a non-adaptive consequence of the evolution of male ornamentation [2,3], but several recent studies show that females can also benefit from expressing elaborate ornaments [reviewed in [4-8]]. Therefore, female ornaments appear to be adaptive in many cases.

A little addressed question is whether female ornaments are adaptations *stricto sensu *(i.e., evolved due to selection on females). This is an important question because current utility of a trait does not imply evolutionary causation [9-11]. In the case of female ornaments, it was suggested that they, and the mechanisms that make them adaptive (e.g., male mate preferences), could evolve as pleiotropic consequences to selection on the opposite sex, i.e., sexual constraint [3,12-14]. We use the word "constraint" in the sense of a genetic phenomenon biasing evolution towards certain phenotypes but not absolutely barring others, rather than in the stronger sense of "evolutionary forbidden trajectories" or "absolute evolutionary constraints" [15]. One way of addressing this question is to investigate the historical pattern of evolution of female ornaments relative to male ornaments. This was done in different taxa (insects, cichlid fishes, herptiles and birds) with mixed results [16-24]. All the existing studies reconstructed ancestral states for male and female ornaments. Some of them [16,17] found that male and female ornaments originated asynchronously, suggesting that female ornaments did not evolve by sexual constraint but rather due to selection on females. Alternatively, it could also be that asynchronous evolution of male and female ornamentation reflects selection on female traits (e.g., aggressiveness) that are developmentally linked to ornaments, rather than selection of female ornaments *per se *[24]. These results should be interpreted with caution because ancestral state reconstruction is imprecise [25-27], and it produces wide confidence intervals [28,29] especially when analysing evolutionarily labile traits, as is typical for sexual ornaments [30,31]. Investigating the synchrony of male and female ornament evolution using ancestral state reconstruction should be particularly sensitive to this problem. This is because when male and female ornamentation evolved synchronously reconstruction inaccuracies desynchronise the inferred changes, but when evolution was asynchronous such inaccuracies will probably still yield a pattern of asynchronous evolution (since there are many ways to be asynchronous). This can bias conclusions towards asynchronous, and thus independent, evolution of female ornamentation.

Alternative comparative methods are therefore needed for a better understanding of the relation between the evolution of male and female ornaments. In this paper we use a method based on evolutionary simulations to investigate the evolution of male and female coloration of *Carduelis *finches (Aves, Fringillidae, Carduelinae). Briefly, we simulated male and female evolution superimposing alternative models that can cause the observed correlation between the sexes. The uncertainties of the evolutionary process that hamper ancestral state reconstructions are not discarded here, but are incorporated in the simulations in the form of evolution by random motion (e.g., [32,33]). This noise is used to establish confidence intervals for the predictions of the different evolutionary models, which are then compared to the real data.

We simulated three simple models for the evolution of male and female coloration (which we call constraint on females, constraint on males, and mutual constraint) and test their predictions against a real dataset. In the model of constraint on females, males evolve independently and the similarity between the sexes is entirely due to the evolution of females being constrained by the conspecific male phenotypes. The model of constraint on males is the exact reverse: the similarity between the sexes is due to male phenotypes being constrained by the conspecific female phenotypes. These are very simple models but, since they are the exact reverse of each other, they provide an unbiased framework to test whether constraint on males or on females was the most important evolutionary mechanism. Finally, the model of mutual constraint simulates an intermediate situation, where evolution in both sexes is constrained by the phenotype of the other sex. The important feature of this last model is its symmetry, whereby the phenotypes of males and females are equally pulled towards each other.

We test these models against data from a single large genus of birds, because the coloration of closely related species is expected to exhibit similar patterns of organization [34,35], and thus it is likely that we are quantifying coloration traits that are homologous across species. Most species in the genus *Carduelis *have both yellow or red carotenoid-based coloration [36-39], and a variable extent of black melanin-based coloration that ranges from the wing coverts to almost the entire body [40]. *Carduelis *comprises both sexually monochromatic species (i.e., where males and females look alike) and sexually dichromatic species with males having more ornamental coloration than females, but no apparent cases of "reversed" sexual dichromatism [40]. There is evidence that in this genus carotenoid coloration is object of female preferences [41,42], a better indicator of condition than melanin coloration [42,43], and more evolutionarily labile [44]. This suggests that in this group sexual selection influences the evolution of carotenoid coloration, maybe more strongly than melanin coloration [45]. For sexually selected traits the optimal male and female phenotypes are more likely to differ, because males can be subject to stronger sexual selection due to their greater variance in reproductive success [46]. Therefore, we hypothesised that sexual constraint may be more important for carotenoid coloration because of the putatively different phenotypic optima for males and females.

## Methods

### Coloration measurements

We measured 26 coloration traits (including colours, extent of colour patches, and pigmentation patterns) on skins of the 29 *Carduelis *spp. available at the ornithological collection of the Natural History Museum of London. Detailed descriptions of measurements, as well as the male and female values for each species, are given in ref. [47]. Briefly, we obtained reflectance spectra for various body parts where carotenoid- and melanin-based plumage coloration (i.e., greenish-yellow to red coloration, and dark-grey to black, respectively [36-39,48]) are expressed consistently across species [40], and computed measures of colour brightness, saturation and, for carotenoid-based coloration, hue. Plumage colours of museum specimens can fade with time, but for good quality specimens that is too slight to invalidate their use in studies of avian coloration (e.g., [49,50]), especially for comparisons between sexes and species, as we do here. We also quantified the extension of carotenoid and melanic coloration in various body parts, and categorized the patterns of pigmentation and the colour of beak and legs.

In order to obtain inclusive measures of ornamental coloration, we calculated scores for different types of coloration (carotenoid-based, melanin-based, and all coloration) as described in ref. [47]. Briefly, for carotenoid coloration we first ran a Principal Component Analysis (PCA) on the set of 16 measurements obtained from the areas where *Carduelis *species most often have carotenoid coloration (breast, wing, tail featheredges and rump). This returned three Principal Components (PCs) that are significant by the broken-stick criterion [51], and together they explain 61% of the variation. We calculated the carotenoid coloration score as the sum of these PC scores scaled by their eigenvalues. Similarly, for melanin coloration we ran a PCA on the four measures derived from black coloration. The eigenvalue of the first PC from these PCA is lower than the critical broken-stick value, but the eigenvalues of the first two PCs are larger than one and explain 65% of the variation. Thus, we computed the melanin coloration score as the sum of these two PC scores scaled by their eigenvalues. These PCs are characterised by several strong positive trait loadings of the coloration measurements and few and weak negative loadings, and therefore quantify different aspects of ornamental elaboration. Finally, we obtained an overall score for all coloration measurements from a PCA on all 26 measurements. This returned five PCs with eigenvalues that are significant by the broken-stick criterion, and together they explain 66% of the variation. As above, these PCs are characterised by strong positive trait loadings, and negative loadings are much fewer and lower in absolute value. We computed the total coloration score as the sum of those five PC scores scaled by their eigenvalues. Trait loadings for all PCs are given in the online Supplementary Table S2 of ref. [47].

We looked primarily at the evolution of these three coloration scores, because they quantify ornamentation in a comprehensive way. Although individual PCs explain only a small proportion variation in coloration, we also analysed the first PC (PC1) of each coloration type separately, to assess if results are qualitatively different when studying composite coloration scores or simple PCs.

### Phylogeny reconstruction

We reconstructed the phylogeny of the genus by Bayesian inference with the software MrBayes 3.1 [52,53] using all the *Carduelis *and outgroup mitochondrial cytochrome b sequences in ref. [54]. We obtained the consensus tree using a General Time Reversible model with a proportion of invariable sites and gamma-distributed rate variation across sites (to account for multiple nucleotide substitutions in the same sites), and running it until trees converged (standard deviation of split frequencies < 0.01 at 3 million generations). The resulting phylogeny is given in ref. [47]. The phylogenetic tree comprises 23 of the species in our dataset of colour measurements, and we based our subsequent phylogeny-based analysis on this subset of species. We found that the coloration of *Carduelis*, both male and female, and both carotenoid and melanin, fits a speciational tree significantly better than a chronogram or a genetic distances tree [47]. Therefore, we used the rooted speciational tree in all analyses (speciational tree in Figure 1B of ref. [47]).

**Figure 1:**
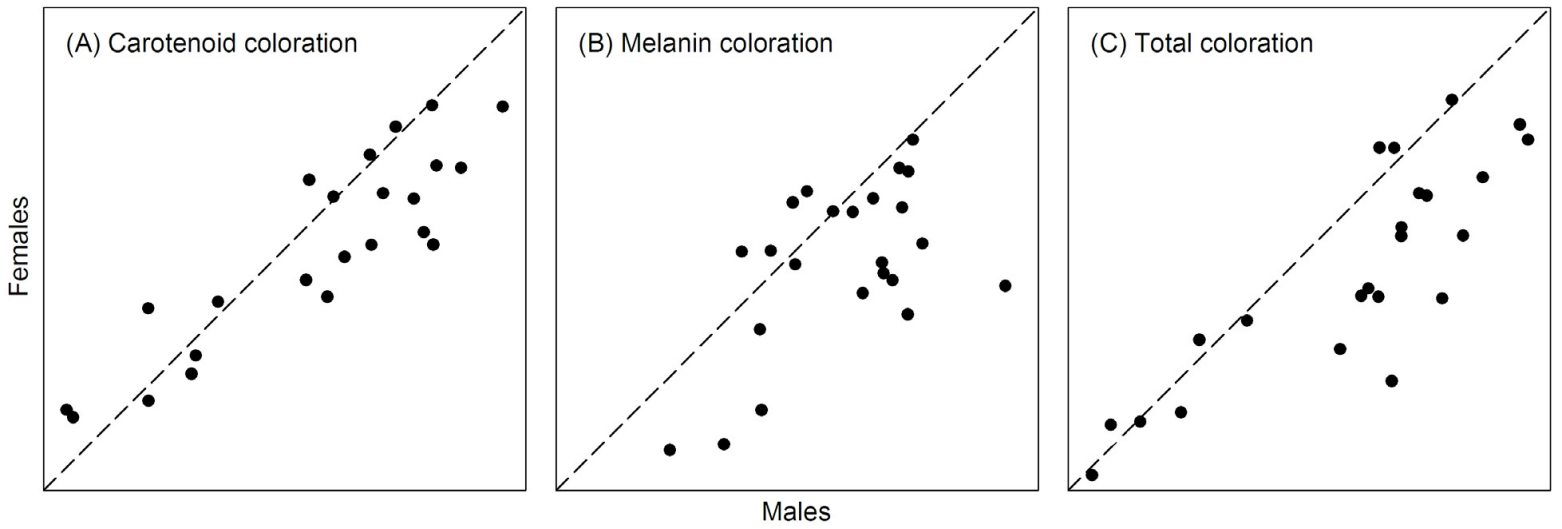
**Male and female coloration scores**. Relation of male and female scores for carotenoid coloration (A), melanin coloration (B) and total coloration (C). Dashed diagonals represent perfect sexual monochromatism (1:1 lines). Species at the lower left area of the plot are sexually monochromatic and little ornamented, at the upper right area are monochromatic and very ornamented, and towards the lower right area are dichromatic with males more ornamented than females.

### Simulations

The following procedure simulates different possible causes for the similarity between males and females. Therefore, all evolutionary simulations are calibrated to produce the real correlation observed between the sexes in the 23 species of *Carduelis *comprised in the phylogeny. The correlation coefficient between the sexes is different for the different types of coloration. Thus, we ran separate simulations, each calibrated to produce on average the real correlation coefficient observed for the appropriate type of coloration.

In all simulated models, Brownian motion makes the sexes evolve independently of each other. Brownian motion was generated by adding a random number, from a normal distribution with average zero and standard deviation one, stepwise for each branch in the phylogeny from root to tips (Table 1, step 1; e.g., refs. [32,33]). The standard deviation of the random numbers is arbitrary: results are identical as long as it is the same in all simulations. This provides synchronous phenotypes of males and females at each node in the phylogeny, which we modified by one of three forms of constraint (Table 1, steps 2 and 3). The three forms of constraint that we simulated all avoid reversed sexual dichromatism by preventing male scores being lower than females' (or, equivalently, preventing female scores being higher than males'; Table 1, step 2), and generate correlated evolution between the sexes by approaching the male and female conspecific scores in each lineage (Table 1, step 3). The assumption that male coloration scores are higher or equal to females' (Table 1, step 2) is necessary because otherwise we would obtain both sexually dichromatism and reversed sexual dichromatism, which is unrealistic because there is no reversed sexual dichromatism in *Carduelis *(i.e., female coloration is similar to or less conspicuous than males', Figure 1; ref. [40]). Like in many other taxa, the preponderance of sexual dimorphism in which males are the more ornamented sex is thought to reflect the generally higher intensity of sexual selection on males [46]. This step was simulated in a very simple way but, since in opposite models this step is exactly reversed, it does not introduce any bias in the testing procedure. Likewise, the approach of the conspecific male and female scores (Table 1, step 3) is necessary so that the extant male and female phenotypes are correlated, and this too was applied to opposite models in a reversed way that does not introduce bias.

**Table 1:** Evolutionary simulations.

Model	Step 1: Brownian motion	Step 2: Block reversed sexual dichromatism	Step 3: Simulate constraint
Constraint on females	M = M_P _+ R	M = M_P_	M = M_P_
	F = F_P _+ R	if F_P _> M_P_, then, F = M_P_, else, F = F_P_	F = F_P _+ C*(M_P _- F_P_)

Constraint on males	as above	F = F_P_	F = F_P_
		if F_P _> M_P_, then, M = F_P_, else, M = M_P_	M = M_P _+ C*(F_P _- M_P_)

Mutual constraint	as above	if F_P _> M_P_, then, M = (M_P _+ F_P_)/2, and F = (M_P _+ F_P_)/2,	M = M_P _+ C*(F_P _- M_P_)
		else, M = M_P_, and F = F_P_	F = F_P _+ C*(M_P _- F_P_)

Formulae in the simulation algorithms for the three evolutionary models. These steps are run consecutively for each branch of the phylogeny, and the simulations repeated 1000 times. M: male phenotype, F: female phenotype, R: random number from a standardized normal distribution, C: constant that calibrates each model to produce simulated extant phenotypes with an average correlation between the sexes equal to the correlation of real data, P: denotes previous step (the previous step of step 1 is the last step in the previous branch of the phylogeny). See text for details.

These constraints (Table 1, steps 2 and 3) affect only one or both sexes in the different models. In the model of constraint on females this affects only the females' phenotypes, in the model of constraint on males this affects only the males', and it affects both sexes equally in the mutual constraint model, as follows:

In the model of constraint on females, females cannot surpass the synchronous conspecific male score in any time interval (when that happens the female score is reset to be equal to the male's), and at each step in the phylogeny, the female phenotype is pulled in the direction of the conspecific male by a constant proportion of their phenotypic difference. Likewise, in the model of constraint on males, male scores cannot be lower than the conspecific female scores (when that happens the male score is automatically reset to be equal to the female's), and are pulled towards the conspecific female phenotype by a constant proportion of phenotypic differences. In the model of mutual constraint, when the male is less conspicuous than the female they are both set to their average; also, both sexes are equally pulled to the average of their phenotype values by a constant proportion of their phenotypic difference. The constant used to approach the phenotypes of the two sexes in each model (Table 1, step 3) was calculated iteratively so as to produce extant phenotypes with an average correlation between extant males and females equal to the real *Carduelis *data. The models are thus equally parameterised, allowing for direct comparison of their predictions [55].

### Statistical testing

Each of the above simulations was run 1000 times, and from the set of extant phenotypes produced in each run we computed three descriptive statistics. These are the statistics that will be compared with the real *Carduelis *data, and we predicted *a priori *that these would differ between models. The statistics were computed after standardization of the joint distribution of male and female extant phenotypes. The three descriptive statistics are: 1) the difference of standard deviations of male and female extant phenotype distributions, as the dispersion of phenotypes is expected to be lower in the constrained sex because constraints limit the extent of independent evolution; 2) the skew (Zg_1 _of D'Agostino and Pearson [56], in ref. [57], p. 117) of the combined distribution of male and female extant phenotypes, as this distribution is predicted to be asymmetrical in the direction of the more variable independent sex; and 3) the difference of slopes of the regressions between the sexes' phenotypes, which is predicted to vary depending on which sex is constrained.

Details of the rationale and predictions for these statistics are as follows. 1) The dispersion of phenotypes is expected to be lower in the constrained sex because the modelled constraints impose limits to evolutionary diversification. Thus, the difference of male and female standard deviations tends to be positive when males are the independent sex, negative when females are the independent sex, and intermediate between those in the mutual constraint model. 2) The joint distribution of simulated phenotypes is made up of two distributions (male and female) with different averages. As the phenotypes of the independent sex tend to be more variable, the joint distribution will tend to be asymmetric and skewed in the direction of the independent sex; in the mutual constraint model skews should be intermediate. 3) The slope of the regression of female on male phenotypes is expected to be lower when males are the independent sex and higher when females are the independent sex. This is because in this group females are ornamented to a similar or lesser degree than males. Thus, when males are the independent sex, any decrease in male coloration of sexually monochromatic species pulls the female phenotype towards the lower left area of the plots in Figure 1 (i.e., sexually monochromatic and little ornamented species), increasing the density of species there and resulting in shallower slopes. If females were the independent sex, decreasing female ornamentation would not result in a similar increase in the density of sexually monochromatic and little ornamented species, because male coloration might or might not also decrease. On the contrary, steeper slopes are expected when females are the independent sex, because increasing female coloration of sexually monochromatic species pulls male phenotypes to the upper right area of the plots (i.e., monochromatic and ornamented species). If males were the independent sex, increasing male ornamentation would not result in a corresponding increase in the density of these sexually monochromatic and very ornamented species, because female coloration might or might not also increase. As these asymmetries do not exist in the mutual constraint model, intermediate slopes are expected there. The slope of the regression of female on male phenotypes varies in the opposite direction of the alternative slope of male on female phenotypes; these two slopes show greater sensitivity at one or the other ends of the slope range, so that greater distinctiveness of model predictions is achieved with the difference of the two slopes, which we used.

Using only the simulation results, not the real *Carduelis *data yet, we quantified how distinct are the predictions of each pair of models, as the probability of a statistic generated by a model falling within the 95% confidence interval of the other. This is the probability of statistical type II error when trying to assign a statistic to a single model (the null hypothesis being that the statistic can be assigned to both models) or, equivalently, 1 minus the statistical power to discriminate among models. We conservatively used two-tailed 95% confidence intervals throughout. Finally, we compared each descriptive statistic of the real *Carduelis *standardized data with the distributions generated by the simulated models. A model is rejected when the *Carduelis *data falls outside the two-tailed 95% confidence interval of the model's simulated outcomes. The best support for an evolutionary model implies both not rejecting it, and rejecting its alternatives.

## Results

Figure 1 shows the relation between conspecific male and female carotenoid, melanin and total coloration scores. Male scores are either larger than females' (sexually dichromatic species with males more ornamented than females), or the scores of both sexes are similar (sexually monochromatic species). The correlation between conspecific male and female coloration scores was stronger for carotenoid coloration (r = 0.893, N = 23, P < 0.001) and total coloration (r = 0.848, N = 23, P < 0.001), and weaker for melanin coloration (r = 0.560, N = 23, P = 0.005). Similar results were obtained when controlling for relatedness with phylogenetic General Least Squares (GLS) regressions [58] of female on male coloration scores (GLS regressions run with BayesTraits, available from http://www.evolution.rdg.ac.uk, estimating the parameter λ to quantify and correct for the phylogenetic signal in the data [59]). For carotenoid coloration the standardized GLS regression β = 0.835, for melanin coloration β = 0.638, and for total coloration β = 0.885 (all P < 0.001), and in all cases there was a strong phylogenetic signal (estimated λ for carotenoid coloration = 0.87, for melanin coloration = 0.96, and for total coloration = 0.60).

The predictions of the three evolutionary models were very similar using one of the descriptive statistics: the skew of the joint male and female distribution (SK). The probability of a simulated SK value falling within the confidence interval of an alternative model was always larger than 87%. Therefore, the power to discriminate models with this statistic was low, and it never discriminated among models or rejected any model (Table 2). For the other two statistics [difference of standard deviations (DSD) and difference of regression slopes (DRS)], in the carotenoid coloration and total coloration simulations the power to discriminate between the two extreme models was good (probability of statistics within confidence interval of alternative model always < 42%), but the power to discriminate between the model of mutual constraint and the others was lower (probability of statistics within confidence interval of alternative model always > 69%). For the simulations of melanin coloration, which were calibrated to produce weaker correlations between the sexes, the predictions of each model were more variable and thus the power to discriminate models was low (probability of statistics within confidence interval of alternative model always > 76%). The probabilities of type II error for each pair of models and coloration score are given in the Additional file 1: Supplemental Table S1.

**Table 2:** Percentiles of *Carduelis *data relative to the simulations' predictions.

	Constraint on females	Constraint on males	Mutual constraint
**Carotenoid coloration**			
Difference of standard deviations	0.88 (0.25)	> 0.99 (**< 0.01**)	0.99 (**0.01**)
Skew	0.12 (0.25)	0.20 (0.40)	0.16 (0.31)
Difference of regression slopes	0.07 (0.13)	< 0.01 (**< 0.01**)	< 0.01 (**< 0.01**)

**Melanin coloration**			
Difference of standard deviations	0.18 (0.36)	0.67 (0.67)	0.39 (0.79)
Skew	0.051 (0.10)	0.14 (0.29)	0.09 (0.18)
Difference of regression slopes	0.72 (0.56)	0.49 (0.97)	0.65 (0.70)

**Total coloration**			
Difference of standard deviations	0.55 (0.90)	0.99 (**0.02**)	0.92 (0.16)
Skew	0.11 (0.23)	0.24 (0.48)	0.17 (0.35)
Difference of regression slopes	0.43 (0.87)	0.01 (**0.01**)	0.09 (0.18)

Percentiles of the *Carduelis *coloration data relative to the distribution of each models' predictions and, in parenthesis, corresponding two-tailed P values for rejecting each of the models. Model rejection at the 0.05 α is signalled in bold typeface.

Table 2 shows the percentiles on which the *Carduelis *data fall relative to the predictions of the alternative models. For carotenoid coloration, the *Carduelis *data fall within the interval of confidence of the model of constraint on females for all statistics, and both the models of constraint on males and of mutual constraint were rejected as explaining the similarity between the sexes (statistics: DSD and DRS, both P < 0.01; Table 2). For melanin coloration the *Carduelis *data falls within the confidence intervals of all models (Table 2), and thus there was no model discrimination. For total coloration the results are intermediate between those for carotenoids and melanins: the models of constraint on females and of mutual constraint were not rejected, and the model of constraint on males was rejected (DSD and DRS, P = 0.02 and 0.01, respectively; Table 2).

Supplemental Table S2, in Additional file 1, shows the result of similar analyses using only the PC1 for each type of coloration. For carotenoid coloration, the model of male constraint was rejected using the same statistics as above (DSD and DRS, both P < 0.01), but there was only a non-significant trend to reject the model of mutual constraint (DSD and DRS, P = 0.11 and 0.12). As above, there was no model discrimination for melanin coloration (Additional file 1: Supplemental Table S2). For the PC1 of total coloration there was a non-significant trend for rejecting the model of constraint on males by the same statistics as above (DSD and DRS, P = 0.16 and 0.17). Also for the PC1 of total coloration, SK fell below the lower 5th percentile for all models (Additional file 1: Supplemental Table S2) which, together with the low power of SK to discriminate models, is best interpreted as a poor fit of the simulations to the SK of this PC, rather than model discrimination. Overall, restricting the analysis to PC1 scores yielded less significant results than the ones obtained with the more comprehensive coloration scores, but showed trends in the same directions. Therefore, using the more comprehensive coloration scores improved the clarity of results, rather than causing qualitatively different ones.

## Discussion and Conclusions

Male and female coloration were positively correlated across the genus *Carduelis*. This simple result (male and female conspecifics look alike) is enough to indicate that some mechanism binds the evolution of male and female coloration, but it does not elucidate which mechanisms are responsible for this. Using simulations of evolutionary models for the correlated evolution of male and female phenotypes, we found that for carotenoid coloration this mechanism is asymmetric, affecting female rather than male evolution. The model of sexual constraint on females was the only that explained the data on carotenoid coloration of *Carduelis*, while the predictions of alternative models were rejected. For melanin coloration none of the simulated models was rejected, suggesting that all can potentially explain its evolution. The correlation between the sexes was also weaker for melanin coloration suggesting that, relative to the range of variation in melanin coloration, males and females evolved independently to a greater extent. As a consequence of these differences in the mode of evolution of carotenoid and melanin coloration, the result for the total coloration score, which encompasses both types of coloration, was intermediate between the above. Evolution of coloration across the genus is the sum of evolutionary events over time and several lineages, and therefore different mechanisms can concurrently contribute to the similarity between the sexes. Even for carotenoid coloration, for which the models of constraint on males and mutual constraint were rejected, these mechanisms can have occasional effects. Their contribution to the similarity between the sexes, however, must be small in comparison with sexual constraint on females.

Elaborate coloration, and carotenoid coloration in particular, is usually sexually selected [1,45], and there is evidence that carotenoid coloration in *Carduelis *is a signal of condition and is subject to female preferences [41-43,60-62]. Sexual selection can act both in males and females [4,5,7,8], but in most cases it should be stronger in males because their variance in mating success is typically larger [46]. Therefore, for sexually selected traits the phenotypic optima for males and females often differ [63]. In these situations, classic population genetic models for the evolution of genetically correlated traits [3,64] predict that the more strongly selected trait (in this case, male coloration) evolves largely independently, and that the less strongly selected trait (in this case, female coloration) evolves in a two-step process: first due to genetic correlations with the more strongly selected trait, and eventually towards its adaptive optimum through acquiring genetic and developmental independence. Our result for carotenoid coloration in *Carduelis *agrees with this sexual constraint explanation. This explanation is also supported by long-standing evidence from birds in general. For example, female ornaments in many species are vestigial or incomplete, in cases resembling juveniles [2,65], and oestrogen-dependent sexual dichromatism is common in birds [66], suggesting that the evolution of elaborate coloration initially affected both sexes and was later lost in females [67].

Evolution by sexual constraint used to be regarded as non-adaptive or even maladaptive for females, but this needs not be so. Sexual constraint can cause maladaptive evolution [e.g., [68,69]], but it may also lead to the expression of correlated traits that are neutral or even beneficial in the constrained sex [70,7]. Since female ornaments appear to be adaptive in many cases [reviewed in [4,5,7,8]], these latter outcomes should be common. Therefore, our result does not imply that female carotenoid coloration in *Carduelis *is maladaptive. It simply indicates that the evolution of carotenoid coloration and its evolutionary diversification across this genus were mainly male-driven.

The correlation between the sexes is strong but not perfect, meaning that an amount of independent evolution also occurred, i.e., the evolution of sexual dichromatism. The correlation between the sexes was weaker for melanin than carotenoid coloration. This was unexpected since in passerines sexual dichromatism is mostly due to carotenoid coloration ([44,71,72], but see also [73]), but correlation coefficients are also affected by the overall range of variation in the traits, which may not be comparable for these two types of coloration. The lower correlation for melanin coloration may be due to testosterone modulating the extent of its expression [74,75], and therefore providing enhanced independence of development pathways between males and females. It is possible that overall the amount of independent evolution that occurred was enough to bring female phenotypes to their adaptive optima. In fact, sexual dichromatism is evolutionarily labile in birds [76,77,22], suggesting that the genetic correlations between the sexes can be overcome when female coloration is selected to diverge.

We conclude that the evolution of female carotenoid coloration in *Carduelis *finches, but possibly not of melanin coloration, was mostly driven by male evolution. Since in this group carotenoid coloration appears to be sexually selected in males, the evolution of female carotenoid coloration may be mostly driven by sexual selection on males. This does not imply that female ornamental coloration is not functional, because evolution by sexual constraint is not contrary to the ornaments also being adaptive in females. It does, however, make it doubtful that, contrary to male ornaments, they qualify as adaptations *stricto sensu *(traits that evolved due to their fitness benefits in females).

Ornamental phenotypes, such as the ones studied here, are not the only class of traits that could evolve by sexual constraint. Sexual constraint could affect the evolution of any trait for which phenotypic optima differ between the sexes (which is often true for traits related with reproduction and life-history) and with strong pleiotropy between the sexes. As with ornamentation, the evolution of developmental independence between the sexes may be effective in resolving some of those sexual conflicts [78,79], but sexual constraint has been suggested to limit the evolution of several non-ornamental traits as well [80], such as body size (e.g., [81-83]), immune defence [84], or levels of circulating hormones [85]. The importance of sexual constraint in the evolution of ornamental and non-ornamental traits must ultimately be settled empirically and, when sexual constraint is predicted to be asymmetrical (i.e. to constrain one sex more than the other), the comparative method we used here may provide a useful tool to evaluate this.

## Authors' contributions

GCC and PGM designed the study and collected the data. GCC analysed the data and wrote the paper. Both authors discussed and approved the final manuscript.

## Supplementary Material

Additional file 1 Supplemental Tables S1 and S2. Click here for file
