# Supplementary material for: Evolution of female carotenoid coloration by sexual constraint in Carduelis finches
Source: BMC Evol Biol. 2010 Mar 25;10:82. doi: 10.1186/1471-2148-10-82 (PMC2865479; doi:10.1186/1471-2148-10-82)
Supplement: Additional file 1 — Supplemental Tables S1 and S2. [file 1471-2148-10-82-S1.PDF]

Supplemental Table S1 - Two-tailed probabilities of type II error for discriminating among each pair of models.

	Constraint on females		Constraint on males		Mutual constraint	
	Constraint on males	Mutual constraint	Constraint on females	Mutual constraint	Constraint on males	Constraint on females
**Carotenoid coloration**						
Difference of standard deviations	0.31	0.73	0.25	0.79	0.72	0.77
Skew	0.92	0.95	0.95	0.97	0.96	0.96
Difference of regression slopes	0.32	0.71	0.23	0.76	0.69	0.78
**Melanin coloration**						
Difference of standard deviations	0.79	0.89	0.76	0.92	0.91	0.93
Skew	0.88	0.96	0.93	0.95	0.95	0.94
Difference of regression slopes	0.91	0.93	0.88	0.92	0.95	0.96
**Total coloration**						
Difference of standard deviations	0.38	0.76	0.32	0.79	0.76	0.80
Skew	0.89	0.95	0.93	0.96	0.96	0.96
Difference of regression slopes	0.42	0.75	0.32	0.79	0.75	0.81

Each cell contains the probability of a prediction from the model on the first line to fall within the two-tailed 95% confidence interval of the alternative model in the second line. Reciprocal values differ slightly because of different shapes of the distributions.

Supplemental Table S2 - Percentiles of the *Carduelis* first principal component score (PC1) for each type of coloration, relative to the distribution of each models' predictions.

	Constraint on females	Constraint on males	Mutual constraint
**PC1 of carotenoid coloration** (31.8%, r = 0.89)			
Difference of standard deviations	0.62 (0.76)	> 0.99 (**0.01**)	0.94 (0.11)
Skew	0.15 (0.31)	0.25 (0.50)	0.20 (0.39)
Difference of regression slopes	0.39 (0.78)	< 0.01 (**< 0.01**)	0.06 (0.12)
**PC1 of melanin coloration** (39.6%, r = 0.60)			
Difference of standard deviations	0.34 (0.68)	0.83 (0.33)	0.62 (0.76)
Skew	0.25 (0.50)	0.51 (0.98)	0.37 (0.75)
Difference of regression slopes	0.50 (> 0.99)	0.22 (0.43)	0.32 (0.63)
**PC1 of total coloration** (26.1%, r = 0.88)			
Difference of standard deviations	0.15 (0.29)	0.92 (0.16)	0.59 (0.83)
Skew	0.01 (**0.03**)	0.03 (0.06)	0.02 (**0.03**)
Difference of regression slopes	0.86 (0.29)	0.09 (0.17)	0.42 (0.84)

Statistics as in Table 2 of main text, and in addition the percentage of variance explained and the real correlation coefficient between the sexes (r) are indicated for each PC1.
